# Supplementary material for: Variation in ant-mediated seed dispersal along elevation gradients
Source: PeerJ. 2019 Apr 11;7:e6686. doi: 10.7717/peerj.6686 (PMC6462393; doi:10.7717/peerj.6686)

Ants Seeds SIBER

Israel Del Toro & Relena R. Ribbons

July, 2018

Table of Contents

## Prepare data and load necessary packages for plotting and GLMM

#May the Code be with You#
setwd('~/Dropbox/Seed Dispersal/Data')

library(vegan)

## Warning: package 'vegan' was built under R version 3.4.4

## Loading required package: permute

## Loading required package: lattice

## This is vegan 2.5-1

library(ggplot2)
library (lme4)

## Warning: package 'lme4' was built under R version 3.4.4

## Loading required package: Matrix

## Warning: package 'Matrix' was built under R version 3.4.4

library(lmerTest)

##
## Attaching package: 'lmerTest'

## The following object is masked from 'package:lme4':
##
## lmer

## The following object is masked from 'package:stats':
##
## step

library(gridExtra)

## Warning: package 'gridExtra' was built under R version 3.4.1

source("summary_se.R")
library (MASS)

## Warning: package 'MASS' was built under R version 3.4.3

library(car)

## Warning: package 'car' was built under R version 3.4.4

## Loading required package: carData

## Warning: package 'carData' was built under R version 3.4.4

library("FactoMineR")
library("devtools")

## Warning: package 'devtools' was built under R version 3.4.3

##
## Attaching package: 'devtools'

## The following object is masked from 'package:permute':
##
## check

library("ggbiplot")

## Loading required package: plyr

## Loading required package: scales

## Warning: package 'scales' was built under R version 3.4.1

## Loading required package: grid

#Overdispersion test function- Modified from B. Bolker July 2018.
overdisp_fun <- function(model) {
 rdf <- df.residual(model)
 rp <- residuals(model,type="pearson")
 Pearson.chisq <- sum(rp^2)
 prat <- Pearson.chisq/rdf
 pval <- pchisq(Pearson.chisq, df=rdf, lower.tail=FALSE)
 c(chisq=Pearson.chisq,ratio=prat,rdf=rdf,p=pval)
}

#load and subset datasets----
data_part<- read.csv('seed_depot_data_10_04_16.csv', header=T)
data_part$Elevation<-as.factor(data_part$Elevation)
data_part$seeds.removed<-as.numeric (data_part$seeds.removed)
str(data_part)

## 'data.frame': 2345 obs. of 15 variables:
## $ Depot.. : int 1 1 1 1 1 1 1 1 1 1 ...
## $ Site : Factor w/ 4 levels "CHI","CNF","LNF",..: 1 1 1 1 1 1 1 1 1 1 ...
## $ Elevation : Factor w/ 3 levels "1600","2200",..: 1 2 3 1 2 3 1 2 3 1 ...
## $ Seed : Factor w/ 4 levels "Datura","Iris",..: 1 1 1 2 2 2 3 3 3 4 ...
## $ X..of.seeds : Factor w/ 29 levels "0","1","10","11",..: 19 19 19 19 29 19 19 19 19 19 ...
## $ Time_cat : int 0 0 0 0 0 0 0 0 0 0 ...
## $ seeds.removed : num 0 0 0 0 0 0 0 0 0 0 ...
## $ hours : int 0 0 0 0 0 0 0 0 0 0 ...
## $ total.seeds.removed: int 0 0 0 NA NA NA NA NA NA NA ...
## $ Lat : num 31.9 31.9 31.9 31.9 31.9 ...
## $ Lon : num -109 -109 -109 -109 -109 ...
## $ Exact.Elevation : int 1602 2206 2787 1602 2206 2787 1602 2206 2787 1602 ...
## $ bare.ground : num 39.9 51.4 55.4 39.9 51.4 ...
## $ t.max : num 47 43 30 47 43 30 47 43 30 47 ...
## $ t.min : num 15 16 10.5 15 16 10.5 15 16 10.5 15 ...

#Partition the dataset by species
datura<-data_part[which(data_part$Seed=="Datura"),]
iris<-data_part[which(data_part$Seed=="Iris"),]
oat<-data_part[which(data_part$Seed=="Oat"),]
sumac<-data_part[which(data_part$Seed=="Sumac"),]

# Use lme4 to create GLMM and test for overdispersion

#GLMM function try with a poisson distribution for count data. Global model
data_part$id <- with(data_part, paste(Site, Elevation, Depot.., sep = "_"))
m1<- glmer(seeds.removed ~ hours + Elevation*Seed+ (1|Site) + (1+hours |id), family= poisson, data_part)

## Warning in checkConv(attr(opt, "derivs"), opt$par, ctrl = control
## $checkConv, : Model failed to converge with max|grad| = 0.103585 (tol =
## 0.001, component 1)

## Warning in checkConv(attr(opt, "derivs"), opt$par, ctrl = control$checkConv, : Model is nearly unidentifiable: very large eigenvalue
## - Rescale variables?

summary(m1)

## Generalized linear mixed model fit by maximum likelihood (Laplace
## Approximation) [glmerMod]
## Family: poisson ( log )
## Formula: seeds.removed ~ hours + Elevation * Seed + (1 | Site) + (1 +
## hours | id)
## Data: data_part
##
## AIC BIC logLik deviance df.resid
## 12224.4 12322.4 -6095.2 12190.4 2328
##
## Scaled residuals:
## Min 1Q Median 3Q Max
## -4.6086 -1.0841 -0.5188 0.4415 10.4497
##
## Random effects:
## Groups Name Variance Std.Dev. Corr
## id (Intercept) 1.7829082 1.33526
## hours 0.0004083 0.02021 -0.91
## Site (Intercept) 0.0364724 0.19098
## Number of obs: 2345, groups: id, 122; Site, 4
##
## Fixed effects:
## Estimate Std. Error z value Pr(>|z|)
## (Intercept) -0.330657 0.185377 -1.784 0.074472 .
## hours 0.056445 0.002139 26.386 < 2e-16 ***
## Elevation2200 -0.517828 0.144649 -3.580 0.000344 ***
## Elevation2800 -0.942897 0.166684 -5.657 1.54e-08 ***
## SeedIris -0.926507 0.050674 -18.284 < 2e-16 ***
## SeedOat 0.149703 0.036815 4.066 4.78e-05 ***
## SeedSumac -1.091200 0.053825 -20.273 < 2e-16 ***
## Elevation2200:SeedIris 0.719278 0.074662 9.634 < 2e-16 ***
## Elevation2800:SeedIris 1.544592 0.087789 17.594 < 2e-16 ***
## Elevation2200:SeedOat 0.866947 0.056303 15.398 < 2e-16 ***
## Elevation2800:SeedOat 1.145066 0.075292 15.208 < 2e-16 ***
## Elevation2200:SeedSumac 0.362698 0.083610 4.338 1.44e-05 ***
## Elevation2800:SeedSumac -0.207741 0.149028 -1.394 0.163327
## ---
## Signif. codes: 0 '***' 0.001 '**' 0.01 '*' 0.05 '.' 0.1 ' ' 1

##
## Correlation matrix not shown by default, as p = 13 > 12.
## Use print(x, correlation=TRUE) or
## vcov(x) if you need it

## convergence code: 0
## Model failed to converge with max|grad| = 0.103585 (tol = 0.001, component 1)
## Model is nearly unidentifiable: very large eigenvalue
## - Rescale variables?

Anova(m1)

## Analysis of Deviance Table (Type II Wald chisquare tests)
##
## Response: seeds.removed
## Chisq Df Pr(>Chisq)
## hours 696.1969 1 <2e-16 ***
## Elevation 0.2394 2 0.8872
## Seed 2652.5446 3 <2e-16 ***
## Elevation:Seed 561.8317 6 <2e-16 ***
## ---
## Signif. codes: 0 '***' 0.001 '**' 0.01 '*' 0.05 '.' 0.1 ' ' 1

#Overdispersion test
overdisp_fun(m1)

## chisq ratio rdf p
## 7568.900321 3.251246 2328.000000 0.000000

#These results indicate the global model is not overdispersed.

###Figure 1 Global model of total number of seeds removed over time.
M1<- summarySE(data_part, measurevar="seeds.removed", groupvars=c("hours","Elevation"))
total.seeds.graph<-ggplot(M1, aes(x=hours , y=seeds.removed, group = Elevation)) +
 geom_errorbar (aes(ymin=seeds.removed-se, ymax=seeds.removed+se), size = 1, width=.3, position=position_dodge(.01)) + theme_bw() +
 geom_line(aes(), size = 1) +
 geom_point(aes(shape = Elevation), size = 6) +
 theme(legend.position=("none")) + theme(text = element_text(size=20)) +
 xlab ("Time") +ylab("Seeds removed")
total.seeds.graph


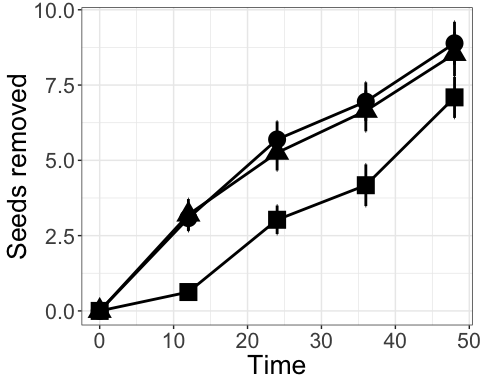


tiff (filename = "Figure1.tiff", height= 4, width= 4, units= "in", res=300)
total.seeds.graph
dev.off()

## quartz_off_screen
## 2

###ANOVA Table
datura$id <- with(datura, paste(Site, Elevation, Depot.., sep = "_"))
Datura.lmer<- glmer (seeds.removed ~ hours + Elevation + Site + (1|Site) + (1+hours|id), family= poisson, datura)

## Warning in checkConv(attr(opt, "derivs"), opt$par, ctrl = control$checkConv, : Model failed to converge with max|grad| = 0.17102 (tol = 0.001, component 1)

## Warning in checkConv(attr(opt, "derivs"), opt$par, ctrl = control$checkConv, : Model is nearly unidentifiable: very large eigenvalue
## - Rescale variables?

summary(Datura.lmer)

## Generalized linear mixed model fit by maximum likelihood (Laplace
## Approximation) [glmerMod]
## Family: poisson ( log )
## Formula: seeds.removed ~ hours + Elevation + Site + (1 | Site) + (1 +
## hours | id)
## Data: datura
##
## AIC BIC logLik deviance df.resid
## 2544.4 2592.7 -1261.2 2522.4 584
##
## Scaled residuals:
## Min 1Q Median 3Q Max
## -3.2959 -0.7552 -0.3380 0.4553 4.9121
##
## Random effects:
## Groups Name Variance Std.Dev. Corr
## id (Intercept) 2.848e+00 1.687460
## hours 3.509e-04 0.018731 -0.87
## Site (Intercept) 2.166e-05 0.004654
## Number of obs: 595, groups: id, 122; Site, 4
##
## Fixed effects:
## Estimate Std. Error z value Pr(>|z|)
## (Intercept) -0.182973 0.296574 -0.617 0.5373
## hours 0.056948 0.003291 17.305 < 2e-16 ***
## Elevation2200 -1.033363 0.238550 -4.332 1.48e-05 ***
## Elevation2800 -1.206025 0.267856 -4.503 6.72e-06 ***
## SiteCNF -0.556846 0.284369 -1.958 0.0502 .
## SiteLNF -0.455368 0.294526 -1.546 0.1221
## SiteMOG 0.157186 0.276616 0.568 0.5699
## ---
## Signif. codes: 0 '***' 0.001 '**' 0.01 '*' 0.05 '.' 0.1 ' ' 1
##
## Correlation of Fixed Effects:
## (Intr) hours El2200 El2800 SitCNF SitLNF
## hours -0.634
## Elevatn2200 -0.429 0.145
## Elevatn2800 -0.460 0.292 0.431
## SiteCNF -0.460 -0.010 0.033 -0.043
## SiteLNF -0.388 -0.061 -0.084 -0.089 0.492
## SiteMOG -0.402 -0.104 -0.003 -0.130 0.515 0.516
## convergence code: 0
## Model failed to converge with max|grad| = 0.17102 (tol = 0.001, component 1)
## Model is nearly unidentifiable: very large eigenvalue
## - Rescale variables?

Anova(Datura.lmer)

## Analysis of Deviance Table (Type II Wald chisquare tests)
##
## Response: seeds.removed
## Chisq Df Pr(>Chisq)
## hours 299.4644 1 < 2.2e-16 ***
## Elevation 27.2981 2 1.181e-06 ***
## Site 9.2003 3 0.02674 *
## ---
## Signif. codes: 0 '***' 0.001 '**' 0.01 '*' 0.05 '.' 0.1 ' ' 1

overdisp_fun(Datura.lmer)

## chisq ratio rdf p
## 7.428158e+02 1.271945e+00 5.840000e+02 8.494260e-06

iris$id <- with(iris, paste(Site, Elevation, Depot.., sep = "_"))
Iris.lmer<- glmer (seeds.removed ~ hours + Elevation + Site + (1|Site) + (1+hours|id), family= poisson, iris)

## Warning in checkConv(attr(opt, "derivs"), opt$par, ctrl = control$checkConv, : Model failed to converge with max|grad| = 0.0247559 (tol = 0.001, component 1)

## Warning in checkConv(attr(opt, "derivs"), opt$par, ctrl = control$checkConv, : Model is nearly unidentifiable: very large eigenvalue
## - Rescale variables?

summary(Iris.lmer)

## Generalized linear mixed model fit by maximum likelihood (Laplace
## Approximation) [glmerMod]
## Family: poisson ( log )
## Formula: seeds.removed ~ hours + Elevation + Site + (1 | Site) + (1 +
## hours | id)
## Data: iris
##
## AIC BIC logLik deviance df.resid
## 2042.8 2090.7 -1010.4 2020.8 568
##
## Scaled residuals:
## Min 1Q Median 3Q Max
## -3.2672 -0.6466 -0.2989 0.3945 3.5110
##
## Random effects:
## Groups Name Variance Std.Dev. Corr
## id (Intercept) 3.164e+00 1.7786582
## hours 3.301e-04 0.0181689 -0.87
## Site (Intercept) 2.395e-07 0.0004894
## Number of obs: 579, groups: id, 122; Site, 4
##
## Fixed effects:
## Estimate Std. Error z value Pr(>|z|)
## (Intercept) -2.396556 0.325212 -7.369 1.72e-13 ***
## hours 0.056782 0.003553 15.983 < 2e-16 ***
## Elevation2200 0.296634 0.272916 1.087 0.27708
## Elevation2800 0.944576 0.270078 3.497 0.00047 ***
## SiteCNF 0.427814 0.321131 1.332 0.18279
## SiteLNF 0.541278 0.342683 1.580 0.11421
## SiteMOG 0.962541 0.320539 3.003 0.00267 **
## ---
## Signif. codes: 0 '***' 0.001 '**' 0.01 '*' 0.05 '.' 0.1 ' ' 1
##
## Correlation of Fixed Effects:
## (Intr) hours El2200 El2800 SitCNF SitLNF
## hours -0.421
## Elevatn2200 -0.413 -0.042
## Elevatn2800 -0.446 -0.010 0.502
## SiteCNF -0.497 -0.081 -0.014 -0.008
## SiteLNF -0.476 -0.154 -0.016 0.030 0.529
## SiteMOG -0.515 -0.151 0.052 0.022 0.554 0.576
## convergence code: 0
## Model failed to converge with max|grad| = 0.0247559 (tol = 0.001, component 1)
## Model is nearly unidentifiable: very large eigenvalue
## - Rescale variables?

Anova(Iris.lmer)

## Analysis of Deviance Table (Type II Wald chisquare tests)
##
## Response: seeds.removed
## Chisq Df Pr(>Chisq)
## hours 255.4472 1 < 2.2e-16 ***
## Elevation 12.8307 2 0.001636 **
## Site 9.1792 3 0.027001 *
## ---
## Signif. codes: 0 '***' 0.001 '**' 0.01 '*' 0.05 '.' 0.1 ' ' 1

#This results suggest an overdispersion effect on the Iris LMER We can still fit is assuming a pseudo Poisson distribution,

overdisp_fun(Iris.lmer)

## chisq ratio rdf p
## 471.9677584 0.8309292 568.0000000 0.9986839

#This model is over dispersed so must be corrected to interpret the potential source of Type 1 error (below):
## extract summary table; you may also be able to do this via
## broom::tidy or broom.mixed::tidy
cc <- coef(summary(Iris.lmer))
phi <- overdisp_fun(Iris.lmer)["ratio"]
cc <- within(as.data.frame(cc),
{ `Std. Error` <- `Std. Error`*sqrt(phi)
 `z value` <- Estimate/`Std. Error`
 `Pr(>|z|)` <- 2*pnorm(abs(`z value`), lower.tail=FALSE)
})
printCoefmat(cc,digits=3)

## Estimate Std. Error z value Pr(>|z|)
## (Intercept) -2.39656 0.29645 -8.08 6.3e-16 ***
## hours 0.05678 0.00324 17.53 < 2e-16 ***
## Elevation2200 0.29663 0.24878 1.19 0.23312
## Elevation2800 0.94458 0.24619 3.84 0.00012 ***
## SiteCNF 0.42781 0.29273 1.46 0.14389
## SiteLNF 0.54128 0.31237 1.73 0.08313 .
## SiteMOG 0.96254 0.29219 3.29 0.00099 ***
## ---
## Signif. codes: 0 '***' 0.001 '**' 0.01 '*' 0.05 '.' 0.1 ' ' 1

#The estimated pseudo-poisson fit suggest that the effect of Iris seen at High elevations is largely driven by the trends at two mountains LNF and MOG

oat$id <- with(oat, paste(Site, Elevation, Depot.., sep = "_"))
Oat.lmer<- glmer (seeds.removed ~ hours + Elevation + Site + (1|Site) + (1+hours|id), family= poisson, oat)

## Warning in checkConv(attr(opt, "derivs"), opt$par, ctrl = control$checkConv, : Model failed to converge with max|grad| = 0.293092 (tol = 0.001, component 1)

## Warning in checkConv(attr(opt, "derivs"), opt$par, ctrl = control$checkConv, : Model is nearly unidentifiable: very large eigenvalue
## - Rescale variables?

summary(Oat.lmer)

## Generalized linear mixed model fit by maximum likelihood (Laplace
## Approximation) [glmerMod]
## Family: poisson ( log )
## Formula: seeds.removed ~ hours + Elevation + Site + (1 | Site) + (1 +
## hours | id)
## Data: oat
##
## AIC BIC logLik deviance df.resid
## 3731.7 3780.0 -1854.8 3709.7 587
##
## Scaled residuals:
## Min 1Q Median 3Q Max
## -3.2997 -1.0421 -0.4422 0.6946 5.8379
##
## Random effects:
## Groups Name Variance Std.Dev. Corr
## id (Intercept) 2.975e+00 1.7247391
## hours 7.345e-04 0.0271018 -0.95
## Site (Intercept) 8.629e-07 0.0009289
## Number of obs: 598, groups: id, 122; Site, 4
##
## Fixed effects:
## Estimate Std. Error z value Pr(>|z|)
## (Intercept) -0.636778 0.221407 -2.876 0.004027 **
## hours 0.062210 0.003066 20.288 < 2e-16 ***
## Elevation2200 0.478713 0.144430 3.314 0.000918 ***
## Elevation2800 0.088885 0.170406 0.522 0.601947
## SiteCNF 0.398577 0.168348 2.368 0.017905 *
## SiteLNF -0.301956 0.186225 -1.621 0.104919
## SiteMOG 0.496104 0.167612 2.960 0.003078 **
## ---
## Signif. codes: 0 '***' 0.001 '**' 0.01 '*' 0.05 '.' 0.1 ' ' 1
##
## Correlation of Fixed Effects:
## (Intr) hours El2200 El2800 SitCNF SitLNF
## hours -0.747
## Elevatn2200 -0.316 -0.012
## Elevatn2800 -0.375 0.100 0.458
## SiteCNF -0.334 -0.051 -0.042 -0.092
## SiteLNF -0.344 0.005 -0.075 -0.047 0.491
## SiteMOG -0.379 -0.023 0.022 -0.030 0.525 0.477
## convergence code: 0
## Model failed to converge with max|grad| = 0.293092 (tol = 0.001, component 1)
## Model is nearly unidentifiable: very large eigenvalue
## - Rescale variables?

Anova(Oat.lmer)

## Analysis of Deviance Table (Type II Wald chisquare tests)
##
## Response: seeds.removed
## Chisq Df Pr(>Chisq)
## hours 411.611 1 < 2.2e-16 ***
## Elevation 12.244 2 0.002194 **
## Site 25.434 3 1.253e-05 ***
## ---
## Signif. codes: 0 '***' 0.001 '**' 0.01 '*' 0.05 '.' 0.1 ' ' 1

overdisp_fun(Oat.lmer)

## chisq ratio rdf p
## 1.246737e+03 2.123913e+00 5.870000e+02 1.170545e-49

sumac$id <- with(sumac, paste(Site, Elevation, Depot.., sep = "_"))
Sumac.lmer<- glmer (seeds.removed ~ hours + Elevation + Site + (1|Site) + (1+hours|id), family= poisson, sumac)

## Warning in checkConv(attr(opt, "derivs"), opt$par, ctrl = control
## $checkConv, : Model failed to converge with max|grad| = 0.268879 (tol =
## 0.001, component 1)

summary(Sumac.lmer)

## Generalized linear mixed model fit by maximum likelihood (Laplace
## Approximation) [glmerMod]
## Family: poisson ( log )
## Formula: seeds.removed ~ hours + Elevation + Site + (1 | Site) + (1 +
## hours | id)
## Data: sumac
##
## AIC BIC logLik deviance df.resid
## 1361.6 1409.4 -669.8 1339.6 562
##
## Scaled residuals:
## Min 1Q Median 3Q Max
## -3.2298 -0.4506 -0.2005 -0.0679 7.4197
##
## Random effects:
## Groups Name Variance Std.Dev. Corr
## id (Intercept) 6.376e+00 2.524979
## hours 1.174e-03 0.034265 -0.83
## Site (Intercept) 3.393e-05 0.005825
## Number of obs: 573, groups: id, 117; Site, 4
##
## Fixed effects:
## Estimate Std. Error z value Pr(>|z|)
## (Intercept) -3.750840 0.594701 -6.307 2.84e-10 ***
## hours 0.076668 0.008824 8.688 < 2e-16 ***
## Elevation2200 0.214430 0.391292 0.548 0.5837
## Elevation2800 -0.929573 0.434201 -2.141 0.0323 *
## SiteCNF 0.109484 0.483950 0.226 0.8210
## SiteLNF 0.944242 0.461876 2.044 0.0409 *
## SiteMOG 0.516605 0.454127 1.138 0.2553
## ---
## Signif. codes: 0 '***' 0.001 '**' 0.01 '*' 0.05 '.' 0.1 ' ' 1
##
## Correlation of Fixed Effects:
## (Intr) hours El2200 El2800 SitCNF SitLNF
## hours -0.729
## Elevatn2200 -0.439 0.163
## Elevatn2800 -0.441 0.162 0.456
## SiteCNF -0.397 -0.003 -0.022 0.120
## SiteLNF -0.422 0.015 -0.004 0.092 0.496
## SiteMOG -0.379 -0.035 0.022 0.011 0.487 0.509
## convergence code: 0
## Model failed to converge with max|grad| = 0.268879 (tol = 0.001, component 1)

Anova(Sumac.lmer)

## Analysis of Deviance Table (Type II Wald chisquare tests)
##
## Response: seeds.removed
## Chisq Df Pr(>Chisq)
## hours 75.4874 1 < 2e-16 ***
## Elevation 7.5148 2 0.02334 *
## Site 5.1807 3 0.15904
## ---
## Signif. codes: 0 '***' 0.001 '**' 0.01 '*' 0.05 '.' 0.1 ' ' 1

overdisp_fun(Sumac.lmer)

## chisq ratio rdf p
## 381.0789984 0.6780765 562.0000000 1.0000000

#This model is over dispersed so must be corrected to interpret the potential source of Type 1 error (below):
## extract summary table; you may also be able to do this via
## broom::tidy or broom.mixed::tidy
cc <- coef(summary(Sumac.lmer))
phi <- overdisp_fun(Sumac.lmer)["ratio"]
cc <- within(as.data.frame(cc),
{ `Std. Error` <- `Std. Error`*sqrt(phi)
 `z value` <- Estimate/`Std. Error`
 `Pr(>|z|)` <- 2*pnorm(abs(`z value`), lower.tail=FALSE)
})
printCoefmat(cc,digits=3)

## Estimate Std. Error z value Pr(>|z|)
## (Intercept) -3.75084 0.48971 -7.66 1.9e-14 ***
## hours 0.07667 0.00727 10.55 < 2e-16 ***
## Elevation2200 0.21443 0.32221 0.67 0.5057
## Elevation2800 -0.92957 0.35754 -2.60 0.0093 **
## SiteCNF 0.10948 0.39851 0.27 0.7835
## SiteLNF 0.94424 0.38033 2.48 0.0130 *
## SiteMOG 0.51660 0.37395 1.38 0.1671
## ---
## Signif. codes: 0 '***' 0.001 '**' 0.01 '*' 0.05 '.' 0.1 ' ' 1

####Figure 2 in the paper (4 panel with each species)
Datura<- summarySE(datura, measurevar="seeds.removed", groupvars=c("hours","Elevation"))
head (Datura)

## hours Elevation N seeds.removed sd se ci
## 1 0 1600 40 0.0000000 0.0000000 0.00000000 0.000000
## 2 0 2200 48 0.0000000 0.0000000 0.00000000 0.000000
## 3 0 2800 46 0.0000000 0.0000000 0.00000000 0.000000
## 4 12 1600 40 3.2250000 4.1786239 0.66069845 1.336389
## 5 12 2200 44 3.2954545 6.2266477 0.93870246 1.893074
## 6 12 2800 38 0.2894737 0.5651068 0.09167242 0.185746

datura.graph<-ggplot(Datura, aes(x=hours , y=seeds.removed, group = Elevation)) +
 geom_errorbar (aes(ymin=seeds.removed-se, ymax=seeds.removed+se), size = 1, width=.3, position=position_dodge(.01)) + theme_bw() +
 geom_line(aes(), size = 1) +
 geom_point(aes(shape = Elevation, colour = Elevation), size = 6) +
 theme(legend.position=("none")) + theme(text = element_text(size=20)) +
 xlab ("Time") +ylab("Seeds removed") +
 annotate("text", x = 22, y = 22, label = "Datura")
datura.graph


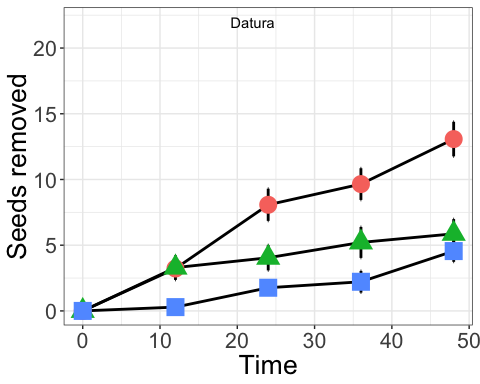


Iris<- summarySE(iris, measurevar="seeds.removed", groupvars=c("hours","Elevation"))
Iris

## hours Elevation N seeds.removed sd se ci
## 1 0 1600 40 0.000000 0.000000 0.0000000 0.0000000
## 2 0 2200 41 0.000000 0.000000 0.0000000 0.0000000
## 3 0 2800 47 0.000000 0.000000 0.0000000 0.0000000
## 4 12 1600 40 1.950000 4.744497 0.7501709 1.5173639
## 5 12 2200 39 2.564103 5.729861 0.9175121 1.8574061
## 6 12 2800 39 1.384615 2.335260 0.3739409 0.7570039
## 7 24 1600 40 3.200000 6.068836 0.9595672 1.9409079
## 8 24 2200 39 3.564103 5.825510 0.9328282 1.8884120
## 9 24 2800 38 3.263158 4.104607 0.6658551 1.3491506
## 10 36 1600 40 3.875000 6.649417 1.0513651 2.1265867
## 11 36 2200 38 4.236842 5.961290 0.9670490 1.9594274
## 12 36 2800 24 3.916667 4.042348 0.8251409 1.7069340
## 13 48 1600 40 4.450000 6.946499 1.0983379 2.2215981
## 14 48 2200 38 5.078947 5.915539 0.9596271 1.9443892
## 15 48 2800 36 7.361111 5.909570 0.9849283 1.9995108

iris.graph<-ggplot(Iris, aes(x=hours , y=seeds.removed, group = Elevation)) +
 geom_errorbar (aes(ymin=seeds.removed-se, ymax=seeds.removed+se), size = 1, width=.3, position=position_dodge(.01)) + theme_bw() +
 geom_line(aes(), size = 1) +
 geom_point(aes(shape = Elevation, colour = Elevation), size = 6) +
 theme(legend.position=("none")) + theme(text = element_text(size=20)) +
 xlab ("Time") +ylab("Seeds removed") +
 annotate("text", x = 22, y = 22, label = "Iris")
iris.graph


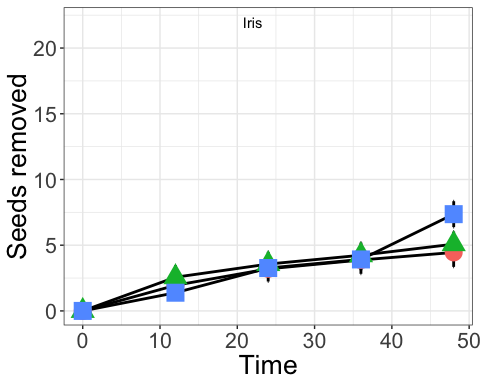


Oat<- summarySE(oat, measurevar="seeds.removed", groupvars=c("hours","Elevation"))
Oat

## hours Elevation N seeds.removed sd se ci
## 1 0 1600 40 0.0000000 0.000000 0.0000000 0.0000000
## 2 0 2200 48 0.0000000 0.000000 0.0000000 0.0000000
## 3 0 2800 47 0.0000000 0.000000 0.0000000 0.0000000
## 4 12 1600 40 5.5750000 6.830878 1.0800567 2.1846208
## 5 12 2200 44 5.7727273 9.258217 1.3957288 2.8147553
## 6 12 2800 39 0.5897436 1.185836 0.1898858 0.3844037
## 7 24 1600 40 8.9750000 8.483733 1.3413959 2.7132293
## 8 24 2200 44 11.4318182 9.458349 1.4258997 2.8756008
## 9 24 2800 39 6.3589744 9.232344 1.4783581 2.9927796
## 10 36 1600 40 10.8000000 8.629704 1.3644760 2.7599133
## 11 36 2200 38 15.1578947 8.011904 1.2997025 2.6334474
## 12 36 2800 25 9.1200000 9.812067 1.9624135 4.0502224
## 13 48 1600 40 14.1750000 8.711582 1.3774221 2.7860992
## 14 48 2200 38 19.0263158 7.674059 1.2448967 2.5224004
## 15 48 2800 36 14.8888889 9.970751 1.6617918 3.3736167

oat.graph<-ggplot(Oat, aes(x=hours , y=seeds.removed, group = Elevation)) +
 geom_errorbar (aes(ymin=seeds.removed-se, ymax=seeds.removed+se), size = 1, width=.3, position=position_dodge(.01)) + theme_bw() +
 geom_line(aes(), size = 1) +
 geom_point(aes(shape = Elevation, colour = Elevation), size = 6) +
 theme(legend.position=("none")) + theme(text = element_text(size=20)) +
 xlab ("Time") +ylab("Seeds removed") +
 annotate("text", x = 22, y = 22, label = "Oat")
oat.graph


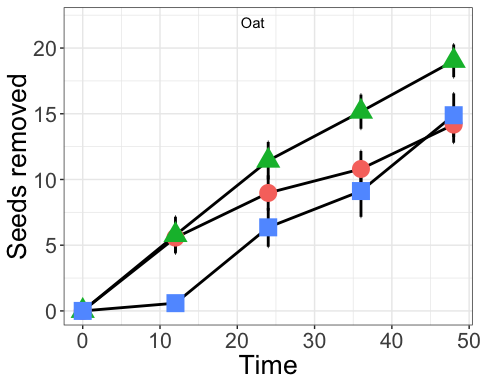


Sumac<- summarySE(sumac, measurevar="seeds.removed", groupvars=c("hours","Elevation"))
Sumac

## hours Elevation N seeds.removed sd se ci
## 1 0 1600 40 0.0000000 0.0000000 0.00000000 0.0000000
## 2 0 2200 48 0.0000000 0.0000000 0.00000000 0.0000000
## 3 0 2800 41 0.0000000 0.0000000 0.00000000 0.0000000
## 4 12 1600 40 1.5750000 4.9711346 0.78600540 1.5898460
## 5 12 2200 44 1.0909091 3.9462563 0.59492052 1.1997716
## 6 12 2800 34 0.1764706 0.4586270 0.07865387 0.1600225
## 7 24 1600 40 2.5250000 5.7154200 0.90368725 1.8278800
## 8 24 2200 44 1.7727273 4.2199065 0.63617484 1.2829688
## 9 24 2800 34 0.3529412 0.6911717 0.11853497 0.2411612
## 10 36 1600 40 3.4750000 6.8086954 1.07654927 2.1775264
## 11 36 2200 38 1.9736842 5.4549102 0.88490329 1.7929844
## 12 36 2800 20 0.5500000 0.8255779 0.18460484 0.3863824
## 13 48 1600 40 3.8500000 6.8296112 1.07985635 2.1842156
## 14 48 2200 38 4.1578947 6.5079009 1.05572093 2.1390938
## 15 48 2800 32 1.0312500 1.4024029 0.24791215 0.5056202

sumac.graph<-ggplot(Sumac, aes(x=hours , y=seeds.removed, group = Elevation)) +
 geom_errorbar (aes(ymin=seeds.removed-se, ymax=seeds.removed+se), size = 1, width=.3, position=position_dodge(.01)) + theme_bw() +
 geom_line(aes(), size = 1) +
 geom_point(aes(shape = Elevation), size = 6) +
 theme(legend.position=c(.8, 0.8)) + theme(text = element_text(size=20)) +
 xlab ("Time") +ylab("Seeds removed") +
 annotate("text", x = 22, y = 22, label = "Sumac")
sumac.graph


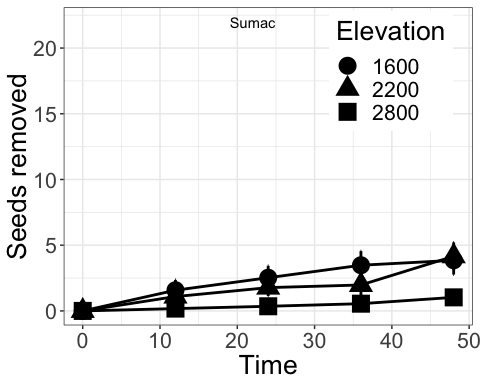


tiff

## function (filename = "Rplot%03d.tiff", width = 480, height = 480,
## units = "px", pointsize = 12, compression = c("none", "rle",
## "lzw", "jpeg", "zip", "lzw+p", "zip+p"), bg = "white",
## res = NA, ..., type = c("cairo", "Xlib", "quartz"), antialias)
## {
## if (!checkIntFormat(filename))
## stop("invalid 'filename'")
## g <- .geometry(width, height, units, res)
## new <- list(...)
## type <- if (!missing(type))
## match.arg(type)
## else getOption("bitmapType")
## if (!missing(antialias))
## new$antialias <- match.arg(antialias, aa.cairo)
## d <- check.options(new, name.opt = ".X11.Options", envir = .X11env)
## antialias <- match(d$antialias, aa.cairo)
## comp <- switch(match.arg(compression), none = 1L, rle = 2L,
## lzw = 5L, jpeg = 7L, zip = 8L, `lzw+p` = 15L, `zip+p` = 18L)
## if (type == "quartz" && capabilities("aqua")) {
## width <- g$width/ifelse(is.na(res), 72, res)
## height <- g$height/ifelse(is.na(res), 72, res)
## invisible(.External(C_Quartz, "tiff", path.expand(filename),
## width, height, pointsize, d$family, d$antialias !=
## "none", "", bg, "white", if (is.na(res)) NULL else res))
## }
## else if (type == "cairo" && capabilities("cairo"))
## invisible(.External(C_devCairo, filename, 8L, g$width,
## g$height, pointsize, bg, res, antialias, comp, d$family,
## 300))
## else invisible(.External2(C_X11, paste0("tiff::", comp, ":",
## filename), g$width, g$height, pointsize, d$gamma, d$colortype,
## d$maxcubesize, bg, bg, d$fonts, res, 0L, 0L, "", 0, 0,
## d$family))
## }
## <bytecode: 0x7f9b869fe758>
## <environment: namespace:grDevices>

grid.arrange(datura.graph, iris.graph, oat.graph, sumac.graph, ncol=2)


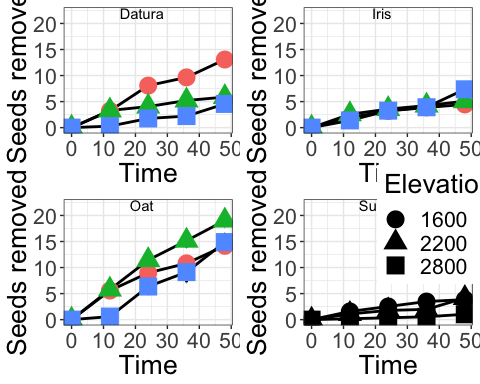


dev.off()

## null device
## 1

df<-read.csv("~/Dropbox/Seed Dispersal/Data/Genera Abundance.csv", header=TRUE)
genera.env<-(df[, c(1:2)])
ant_abund<-df[, c(1,2,18)]
genera<-df[, c(3:17)]
str(genera)

## 'data.frame': 12 obs. of 15 variables:
## $ Pogo : int 16 3 0 15 0 0 10 6 0 2 ...
## $ Formica : int 5 7 0 3 3 0 3 6 4 3 ...
## $ Camponotus : int 0 1 0 0 0 0 0 0 0 0 ...
## $ Crematogaster: int 2 0 0 0 0 0 2 1 0 0 ...
## $ Myrmecocystus: int 12 1 0 0 0 0 9 6 2 1 ...
## $ Tetramorium : int 2 0 0 0 1 0 3 0 0 0 ...
## $ Dorymyrmex : int 15 0 0 0 4 0 9 0 0 0 ...
## $ Forelius : int 2 0 0 0 0 0 3 0 0 0 ...
## $ Pheidole : int 2 0 0 0 2 0 0 0 0 0 ...
## $ Liometopum : int 0 2 0 0 0 0 0 3 0 0 ...
## $ Myrmica : int 0 0 5 0 0 0 0 0 1 0 ...
## $ Aphaenogaster: int 0 0 0 13 0 0 0 0 0 0 ...
## $ Tapinoma : int 0 0 0 0 3 0 0 0 0 0 ...
## $ Monomorium : int 0 0 0 0 3 0 0 0 0 0 ...
## $ Lasius : int 0 0 0 0 0 2 0 0 0 0 ...

# Plot therelationship between abundance and number of seeds removed


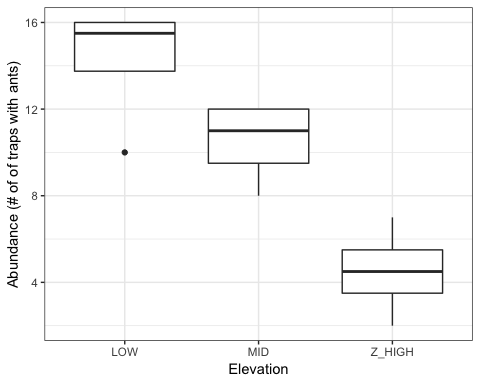


# Principal component analyses of ant genera

cor(genera)

## Pogo Formica Camponotus Crematogaster
## Pogo 1.000000000 0.37649156 -0.09750562 0.6550739
## Formica 0.376491560 1.00000000 0.51796977 0.2742768
## Camponotus -0.097505623 0.51796977 1.00000000 -0.1654758
## Crematogaster 0.655073924 0.27427676 -0.16547585 1.0000000
## Myrmecocystus 0.665630835 0.41303316 -0.13614280 0.9700062
## Tetramorium 0.506654081 0.08155909 -0.15745916 0.8598377
## Dorymyrmex 0.638557341 0.23257195 -0.17522216 0.8292580
## Forelius 0.583073804 0.09551475 -0.13171584 0.9110641
## Pheidole 0.249805456 0.13968606 -0.13483997 0.3436165
## Liometopum -0.002568607 0.62766839 0.50052020 0.1054916
## Myrmica -0.416748436 -0.58527141 -0.14738264 -0.2682709
## Aphaenogaster 0.540713002 -0.04708816 -0.09090909 -0.1654758
## Tapinoma -0.257060280 -0.04708816 -0.09090909 -0.1654758
## Monomorium -0.257060280 -0.04708816 -0.09090909 -0.1654758
## Lasius -0.257060280 -0.47088161 -0.09090909 -0.1654758
## Myrmecocystus Tetramorium Dorymyrmex Forelius Pheidole
## Pogo 0.6656308 0.50665408 0.63855734 0.58307380 0.2498055
## Formica 0.4130332 0.08155909 0.23257195 0.09551475 0.1396861
## Camponotus -0.1361428 -0.15745916 -0.17522216 -0.13171584 -0.1348400
## Crematogaster 0.9700062 0.85983773 0.82925803 0.91106405 0.3436165
## Myrmecocystus 1.0000000 0.77479198 0.84813886 0.83973763 0.3750173
## Tetramorium 0.7747920 1.00000000 0.85357600 0.95818183 0.4670994
## Dorymyrmex 0.8481389 0.85357600 1.00000000 0.83144024 0.6659847
## Forelius 0.8397376 0.95818183 0.83144024 1.00000000 0.2735126
## Pheidole 0.3750173 0.46709937 0.66598470 0.27351263 1.0000000
## Liometopum 0.1408956 -0.22813853 -0.25387488 -0.19083969 -0.1953662
## Myrmica -0.3188119 -0.25527421 -0.28407175 -0.21353891 -0.2186038
## Aphaenogaster -0.2139387 -0.15745916 -0.17522216 -0.13171584 -0.1348400
## Tapinoma -0.2139387 0.15745916 0.08761108 -0.13171584 0.6741999
## Monomorium -0.2139387 0.15745916 0.08761108 -0.13171584 0.6741999
## Lasius -0.2139387 -0.15745916 -0.17522216 -0.13171584 -0.1348400
## Liometopum Myrmica Aphaenogaster Tapinoma
## Pogo -0.002568607 -0.4167484 0.54071300 -0.25706028
## Formica 0.627668387 -0.5852714 -0.04708816 -0.04708816
## Camponotus 0.500520201 -0.1473826 -0.09090909 -0.09090909
## Crematogaster 0.105491627 -0.2682709 -0.16547585 -0.16547585
## Myrmecocystus 0.140895576 -0.3188119 -0.21393869 -0.21393869
## Tetramorium -0.228138531 -0.2552742 -0.15745916 0.15745916
## Dorymyrmex -0.253874883 -0.2840717 -0.17522216 0.08761108
## Forelius -0.190839695 -0.2135389 -0.13171584 -0.13171584
## Pheidole -0.195366166 -0.2186038 -0.13483997 0.67419986
## Liometopum 1.000000000 -0.2135389 -0.13171584 -0.13171584
## Myrmica -0.213538908 1.0000000 -0.14738264 -0.14738264
## Aphaenogaster -0.131715842 -0.1473826 1.00000000 -0.09090909
## Tapinoma -0.131715842 -0.1473826 -0.09090909 1.00000000
## Monomorium -0.131715842 -0.1473826 -0.09090909 1.00000000
## Lasius -0.131715842 -0.1473826 -0.09090909 -0.09090909
## Monomorium Lasius
## Pogo -0.25706028 -0.25706028
## Formica -0.04708816 -0.47088161
## Camponotus -0.09090909 -0.09090909
## Crematogaster -0.16547585 -0.16547585
## Myrmecocystus -0.21393869 -0.21393869
## Tetramorium 0.15745916 -0.15745916
## Dorymyrmex 0.08761108 -0.17522216
## Forelius -0.13171584 -0.13171584
## Pheidole 0.67419986 -0.13483997
## Liometopum -0.13171584 -0.13171584
## Myrmica -0.14738264 -0.14738264
## Aphaenogaster -0.09090909 -0.09090909
## Tapinoma 1.00000000 -0.09090909
## Monomorium 1.00000000 -0.09090909
## Lasius -0.09090909 1.00000000

pca.data= genera
genera.pca = PCA(genera, scale.unit=TRUE, ncp=5, graph=T)


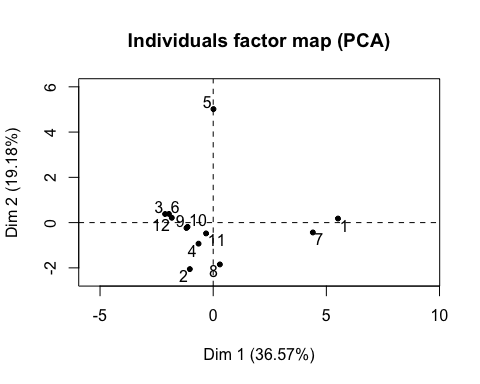

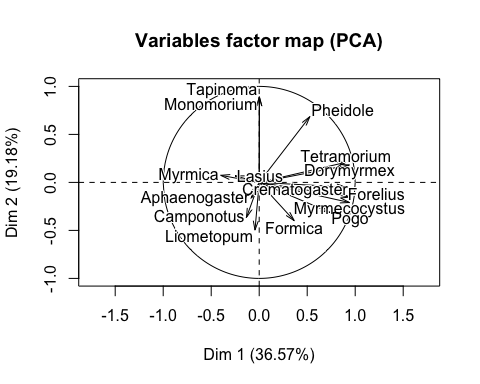


#PCA for coordinate plots
pca <- prcomp(genera, scale. = TRUE)

#PCA with ellipses for Elevation and points for sites.
pca.total.elev <- ggbiplot(pca, obs.scale = 1, var.scale = 1, group=df$Elevation, ellipse = T) +
 scale_colour_manual(values = c("dark green", "blue", "orange")) + scale_shape_manual(values = c(15, 16, 17, 18))+
 geom_point(size = 3, aes(colour=df$Elevation,shape=df$Elevation)) + theme_bw() + theme(panel.grid.minor=element_blank(), panel.grid.major=element_blank()) +
 theme(legend.position="none") + theme(text=element_text(size = 18, colour="black"))
plot(pca.total.elev)


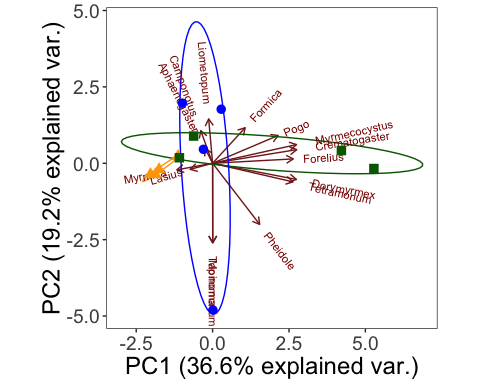

Supplement: Supplemental Information 1 [file peerj-07-6686-s001.docx]
